# Effects of gonadotropin administration on clinical outcomes in clomiphene citrate‐based minimal stimulation cycle IVF

**DOI:** 10.1002/rmb2.12310

**Published:** 2019-12-12

**Authors:** Shinya Karakida, Kenji Ezoe, Junichiro Fukuda, Akiko Yabuuchi, Tamotsu Kobayashi, Keiichi Kato

**Affiliations:** ^1^ Kato Ladies Clinic Tokyo Japan

**Keywords:** clomiphene citrate, cumulative live birthrate, embryo transfer, gonadotropins, in vitro fertilization

## Abstract

**Purpose:**

Exogenous gonadotropins (EGn) have been used occasionally in clomiphene citrate (CC)‐based minimal stimulation cycles to compensate insufficient secretion of endogenous gonadotropin; however, the effectiveness of EGn supplementation remains unknown. In the present study, we assessed whether EGn improved pregnancy outcomes in CC‐based minimal stimulation cycles.

**Methods:**

A total of 223 patients treated with CC and EGn (CC‐EGn group) were matched one to one to patients treated with CC only (CC group) by propensity score matching. Embryonic and pregnancy outcomes were retrospectively compared between the groups.

**Results:**

The numbers of retrieved oocytes, fertilized oocytes, cleaved embryos, and cryopreserved blastocysts were increased in the CC‐EGn group compared with the CC group. However, the cumulative live birthrate was comparable between the two groups. Although the increased number of retrieved oocytes was correlated significantly with improvement of the cumulative live birthrate in both groups, the correlation tended to be lower in the CC‐EGn group than in the CC group (odds ratio, 1.193 vs 1.553).

**Conclusions:**

In CC‐based minimal stimulation cycles, the stimulation should be started with CC only, and EGn administration should be scheduled only if insufficient secretion of endogenous gonadotropin is observed in the late follicular phase.

## 1 INTRODUCTION

Minimal stimulation is an alternative to conventional ovarian stimulation with fewer side effects, such as ovarian hyperstimulation syndrome, and better cost‐effectiveness since the economic burden due to gonadotropin administration and hospital visits is reduced.^1^ Clomiphene citrate (CC) often is administered in minimal ovarian stimulation in vitro fertilization (IVF) treatment.^2^, ^3^, ^4^, ^5^ CC binds hypothalamic estrogen receptors and induces endogenous gonadotropin‐releasing hormone (GnRH) secretion by altering the negative feedback effect of estrogen on the hypothalamus. Clinically, in minimal ovarian stimulation IVF, CC is usually administered alone. However, the minimal necessary follicle‐stimulating hormone (FSH) or human menopausal gonadotropin (hMG) are administered occasionally in the late follicular stage in CC‐based minimal stimulation cycles.^6^ In some cases, multiple follicles develop simultaneously after CC administration. Thus, the serum levels of estradiol, which is produced in the granulosa cells in follicles, substantially increase. The estradiol binds to the estrogen receptors in the hypothalamus and downregulates the secretion of gonadotropins from the pituitary.^7^ If the negative feedback occurs at the mid‐follicular phase, it might lead to insufficient secretion of endogenous FSH and luteinizing hormone (LH), which are required to induce final maturation in the multiple follicles; therefore, exogenous gonadotropins (EGn) have been administered to compensate for the insufficient endogenous FSH and LH secretion, after monitoring serum hormone levels and follicular growth using ultrasound scans, to induce maturation of multiple oocytes.^6^, ^8^ Therefore, administering FSH or hMG in a CC‐based minimal stimulation regimen is considered to improve the oocyte retrieval result followed by IVF outcomes.

Follicle‐stimulating hormone and hMG have been used often for controlled ovarian hyperstimulation (COH) cycles, and the effectiveness of the use of FSH and hMG in COH cycles has been shown worldwide. However, to our knowledge, there are no available studies that have investigated embryonic and pregnancy outcomes in minimal stimulation cycle IVF using CC alone or CC and FSH or hMG. Therefore, in the present study, we retrospectively assessed whether the administration of FSH and hMG improved embryonic and pregnancy outcomes in minimal stimulation cycles.

## 2 MATERIALS AND METHOD

### 2.1 Study patients

**Figure 1:**
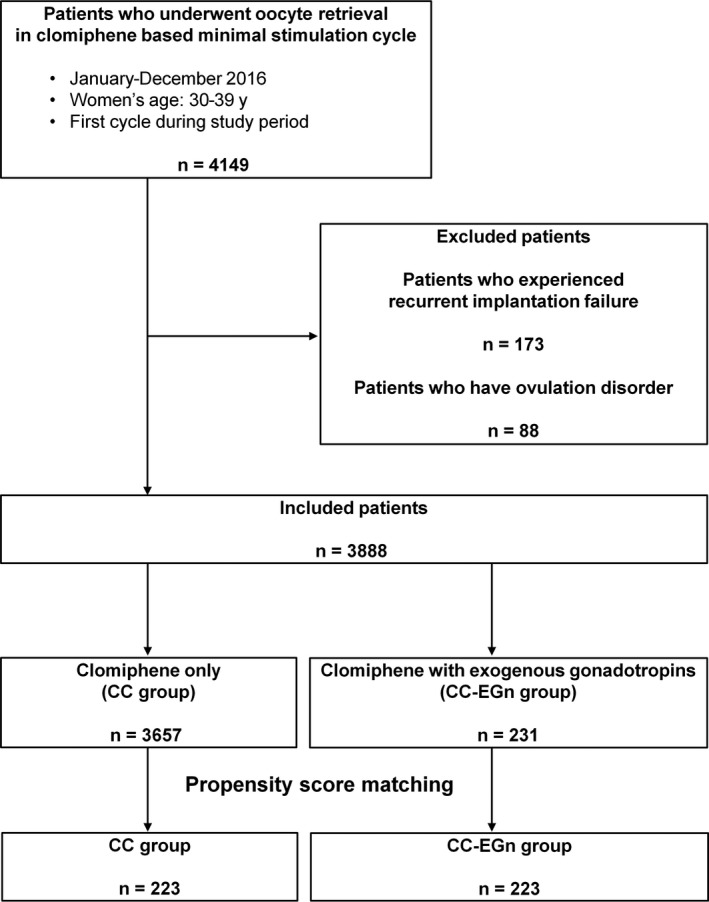
Patient selection flowchart, including inclusion and exclusion criteria. Women who previously underwent embryo transfer four or more times were defined as patients with recurrent implantation failure. CC, clomiphene citrate; EGn, exogenous gonadotropins

In our clinic, assisted reproductive technology is the first choice of any fertility treatment strategy. A total of 3888 patients who underwent oocyte retrieval during CC‐based minimal stimulation cycles between January 2016 and December 2016 at Kato Ladies Clinic were available for analysis, which included 3657 patients who received CC only (CC group) and 231 patients who received CC with EGn (CC‐EGn group). After propensity score matching, 223 patients in the CC‐EGn group were matched one to one to patients in the CC group (Figure 1). The present study only included patients aged 30‐39 years at time of oocyte retrieval. Patients presenting with recurrent implantation failure, that is, those who previously underwent embryo transfer four or more times,^9^ and patients who had ovulation disorder were excluded.

### 2.2 Minimal ovarian stimulation cycle in vitro fertilization

The ovarian stimulation method is usually decided through consultation with patients, with patient preferences taken into account. The detailed protocol for CC‐based minimal stimulation with CC alone has been reported previously.^10^ Briefly, CC (50 mg/d; Fuji Pharma Co., Ltd) was orally administered with an extended regimen, starting on day 3 of the retrieval cycle to the day before induction of final oocyte maturation. Monitoring, which involved an ultrasound scan and hormonal profiles, was usually initiated on day 8 and was performed continuously every other day until the ovulation triggering day. Ovulation triggering was performed using a nasal spray containing the GnRH agonist buserelin (Suprecur; Mochida Pharmaceutical Co., Ltd. or Buserecur; Fuji Pharma Co., Ltd.). A minimal dose of hMG (Ferring Pharma Co., Ltd) or recombinant FSH (rFSH: Merck & Co.) was administered on days 8 and 10 to induce final follicular growth and maturation when (a) there were more than three follicles of uniform size (smaller than 10 mm) in the ovary on the day 8 and (b) the serum FSH level was less than 15 mIU/mL.^6^, ^8^ When the serum FSH level was less than 10 mIU/mL, 150 IU of either hMG or rFSH was administered. When the serum FSH level on day 8 was 10‐15 mIU/mL, 75 IU of hMG or rFSH was administered.

Oocyte retrieval was usually performed 30‐36 hours after triggering using a 21‐G needle (Kitazato Corporation), generally without anesthesia or follicular flushing. Cumulus‐oocyte complexes (COCs) were collected, washed, and then transferred to human tubal fluid (HTF) medium (Kitazato Corporation) with paraffin oil at 5% CO2 in air at 37˚C. These were cultured until use in either conventional IVF (cIVF) 3 hours later or denudation for cases of intracytoplasmic sperm injection (ICSI) 4 hours after oocyte retrieval.^11^ For ICSI, cumulus cells surrounding the oocytes were removed, and the denuded oocytes were cultured in HTF medium covered by paraffin oil for 1 hour before ICSI.

Sperm samples were collected by masturbation and washed by centrifugation through 70% and 90% density gradients (Isolate; Irvine Scientific). Prepared sperm was cultured in HTF medium at 5% CO_2_ in air at 37°C until use.

### 2.3 Conventional insemination, intracytoplasmic sperm injection, embryo culture, and cryopreservation

Conventional insemination or intracytoplasmic sperm injection was performed approximately 3 and 5 hours after oocyte retrieval, respectively.^12^ For cases of cIVF, HTF medium supplemented with 10% serum substitute (Irvine Scientific) was used as a fertilization medium. COCs were cultured with sperm (100 000 sperm/mL) at 5% CO_2_ in air at 37°C. Fertilization assessment was performed 16‐20 hours after insemination. Normally, fertilized zygotes with two pronuclei were individually cultured in the embryo culture medium (Sage One‐step: Origio a/s). In cases of ICSI, oocytes were immediately placed into embryo culture medium, and fertilization was confirmed in the morning of day 1. All embryos were cultured at 37˚C (gas phase: 5% O_2_, 5% CO_2_, and 90% N_2_), in 100% humidity in a water jacket or non‐humidified incubator (Astec Co. Ltd.). The embryos were cultured to the cleavage or blastocyst stage and vitrified for subsequent use in embryo transfer cycles. The embryo vitrification was performed using Cryotop^®^ (Kitazato Biopharma), as described previously.^13^

### 2.4 Embryo transfer

During the study period, single embryo transfers were performed exclusively. Fresh or vitrified‐warmed embryo transfers were performed in spontaneous natural or hormonal replacement cycles as previously described.^6^, ^12^, ^14^, ^15^ Cleaved embryo and blastocyst transfers were performed on days 2‐3 and day 5 after oocyte retrieval or the confirmation of ovulation, respectively. Dydrogesterone (30 mg/d) was routinely orally administered during the early luteal phase after the embryo transfer procedure. In addition, in cases with insufficient luteal function, progesterone was administered intravaginally (Lutinus, Ferring Pharmaceuticals) until the ninth week of pregnancy. The clinical pregnancy rate and ongoing pregnancy rate were defined according to the ultrasonographic observation of a gestational sac at 5‐6 weeks after embryo transfer and observation of fetal heartbeats at 7 weeks after embryo transfer, respectively.

### 2.5 Statistical analyses

In the present study, to reduce the bias of patient characteristics, a propensity score matching was performed using JMP software (SAS). All statistical analyses were performed using JMP software. Continuous parameters were compared via a student's t test. Proportion data were analyzed using the Cochran‐Armitage test for trends and Fisher's exact test. Logistic regression was used to assess the contributing strength of parameters that are potentially associated with pregnancy outcome. Odds ratios (ORs) are reported with 95% confidence intervals (CIs) for each group. A receiver operating characteristic (ROC) analysis also was performed, and the area under the ROC curve (AUC) was calculated. A *P* value <.05 was considered statistically significant.

## 3 RESULTS

### 3.1 Patient characteristics

In this cohort, 3888 patients who underwent oocyte retrieval during CC‐based minimal stimulation cycles were available for analysis (Figure 1). There were 3657 patients who received CC only (CC group) and 231 patients who received CC with EGn (CC‐EGn group). After propensity score matching, 223 patients in the CC‐EGn group were matched one to one to patients in the CC group. Before propensity score matching, some patient characteristics, such as women's age, men's age, infertility cause, and basal estradiol level, varied significantly between the two groups (Table 1). After propensity score matching, the 223 matched pairs were analyzed for differences in patient baseline characteristics (Table 1 and Figure 1). There were no significant differences in the characteristics between the two matched groups.

**Table 1:** Patients' characteristics

	Before propensity score matching			After propensity score matching		
	CC	CC‐EGn	*P* value	CC	CC‐EGn	*P* value
No. of patients, n	3657	231		223	223	
Women's age (years), Mean ± SEM	36.4 ± 0.0	35.3 ± 0.2	<.0001	35.2 ± 0.2	35.3 ± 0.2	.6688
Men's age (years), Mean ± SEM	39.0 ± 0.1	38.4 ± 0.3	.0457	38.1 ± 0.3	38.3 ± 0.3	.6281
Body mass index	20.8 ± 0.0	21.0 ± 0.2	.3115	20.6 ± 0.2	20.9 ± 0.2	.2029
No. of previous OR cycles	0.5 ± 0.0	0.5 ± 0.1	.5301	0.5 ± 0.1	0.5 ± 0.1	.6133
No. of previous ET cycles	0.4 ± 0.1	0.5 ± 0.1	.0957	0.4 ± 0.1	0.5 ± 0.1	.4581
Infertility cause, n (%)						
Oviduct factor	255 (6.9)	11 (4.8)	<.0001	18 (8.1)	11 (4.9)	.5179
Endometrial factor	313 (8.4)	15 (6.5)		20 (9.0)	15 (6.7)	
Male factor	502 (13.7)	30 (13.0)		30 (13.5)	28 (12.6)	
Combination	248 (6.8)	23 (10.0)		17 (7.6)	21 (9.4)	
Unexplained	2339 (64.0)	152 (66.1)		138 (61.9)	148 (66.4)	
Basal serum hormonal level						
Estradiol Mean ± SEM (pg/mL)	36.8 ± 0.3	33.8 ± 1.3	.0289	37.9 ± 1.0	45.3 ± 6.3	.5918
FSH Mean ± SEM (mIU/mL)	10.9 ± 0.1	9.8 ± 0.4	.3257	10.7 ± 0.3	9.7 ± 0.4	.7735

Abbreviations: CC, clomiphene; EGn, exogenous gonadotropins; ET, embryo transfer; FSH, follicle‐stimulating hormone; OR, oocyte retrieval; SEM, standard error of the mean.

### 3.2 In vitro fertilization outcomes

The total dose of CC in the CC group (461.2 ± 6.0 [300‐750] mg) was lower than that in the CC‐EGn group (614.6 ± 8.4 [250‐950] IU) (Table 2). The total EGn dose was 291.9 ± 7.4 [75‐450] IU. The number of oocytes retrieved and inseminated was increased significantly in the CC‐EGn group than in the CC group (Table 2). An increased number of fertilized oocytes and cleaved embryos were obtained in the CC‐EGn group than in the CC group, although fertilization and cleavage rates were statistically comparable between the two groups. Furthermore, the number of cryopreserved blastocysts was higher in the CC‐EGn group than in the CC group (0.6 ± 0.1 vs 1.2 ± 0.1, *P* < .0001). On the other hand, the blastocyst cryopreservation rate was significantly lower in the CC‐EGn group than in the CC group (67.3% vs 58.2%, *P* = .0251).

**Table 2:** In vitro fertilization outcomes in clomiphene cycles with/without exogenous gonadotropin

	CC	CC‐EGn	*P* value
No. of oocyte retrieval cycles	223	223	
Total dose of clomiphene citrate, mg (range)	461.2 ± 6.0 (300‐750)	614.6 ± 8.4 (250‐950)	<.0001
Total dose of EGn, IU (range)	–	291.9 ± 7.4 (75‐450)	–
No. of oocytes retrieved, Mean ± SEM	2.0 ± 0.1	3.7 ± 0.2	<.0001
No. of oocytes inseminated, Mean ± SEM	1.7 ± 0.1	2.8 ± 0.2	<.0001
No. of oocytes fertilized, Mean ± SEM	1.4 ± 0.1	2.6 ± 0.1	<.0001
Fertilization rate, n (%)	319/368 (86.7)	573/615 (93.2)	.0007
No. of cleaved embryos, Mean ± SEM	1.4 ± 0.1	2.5 ± 0.2	<.0001
Cleavage rate, n (%)	314/319 (98.4)	565/573 (98.6)	.8379
No. of cleaved embryos transferred, n	85	76	
No. of cleaved embryos cryopreserved, n	18	27	
No. of blastocysts, Mean ± SEM	0.7 ± 0.1	1.4 ± 0.2	<.0001
Blastocyst formation/Cleaved embryos, n (%)	155/211 (73.5)	318/462 (68.8)	.2229
No. of blastocysts cryopreserved, Mean ± SEM	0.6 ± 0.1	1.2 ± 0.1	<.0001
Blastocyst cryopreservation/Cleaved embryos, n (%)	142/211 (67.3)	269/462 (58.2)	.0251

Abbreviations: CC, clomiphene; EGn, exogenous gonadotropins; SEM, standard error of the mean.

### 3.3 Pregnancy outcomes

The rates of clinical pregnancy, ongoing pregnancy, and live birth after fresh cleaved embryo transfers were comparable between the two groups (Table 3). The live birthrate after a frozen cleaved embryo transfer in the CC‐EGn group was lower than that in the CC group, although there were no differences in the clinical and ongoing pregnancy rates between the groups. The pregnancy outcomes after frozen blastocyst transfer were comparable between the two groups. The cumulative live birthrate in the CC‐EGn group was similar to that in the CC group. Furthermore, we analyzed the correlation between the number of retrieved oocytes and the cumulative live birthrate. In the CC group, the increased number of retrieved oocytes was significantly correlated with improvement of the cumulative live birthrate (OR, 1.553; 95% CIs, 1.223‐1.948; *P* = .0003; AUC, 0.663). The oocyte number in the CC‐EGn group also was associated with the cumulative birthrate (OR, 1.193; 95% CIs, 1.086‐1.325; *P* = .0005; AUC, 0.667). When the cumulative birthrate was stratified by the oocyte number, no significant differences were observed between the groups, although the rates in the CC group were numerically higher than those in the CC‐EGn group (Table S1).

**Table 3:** Pregnancy outcomes after embryo transfer in clomiphene cycles with/without exogenous gonadotropin

	CC	CC‐EGn	*P* value
Fresh cleaved embryo transfer			
No. of transfer cycles	85	76	
Clinical pregnancy, n (%)	28 (32.9)	20 (26.3)	.3589
Ongoing pregnancy, n (%)	25 (29.4)	16 (21.1)	.2242
Live birth, n (%)	22 (25.9)	14 (18.4)	.2567
Frozen cleaved embryo transfer			
No. of transfer cycles	14	15	
Clinical pregnancy, n (%)	7 (50.0)	7 (46.7)	.8575
Ongoing pregnancy, n (%)	7 (50.0)	6 (40.0)	.5884
Live birth, n (%)	7 (50.0)	2 (13.3)	.0329
Frozen blastocyst transfer			
No. of transfer cycles	103	159	
Clinical pregnancy, n (%)	55 (53.4)	80 (50.3)	.6257
Ongoing pregnancy, n (%)	46 (44.7)	74 (46.5)	.7654
Live birth, n (%)	40 (38.8)	59 (37.1)	.7781
Clinical pregnancy/oocyte retrieval, n (%)	88 (39.5)	100 (44.8)	.2497
Ongoing pregnancy/oocyte retrieval, n (%)	78 (35.0)	89 (39.9)	.2818
Live birth/oocyte retrieval, n (%)	69 (30.9)	75 (33.6)	.2177

Abbreviations: CC, clomiphene; EGn, exogenous gonadotropins.

## 4 DISCUSSION

The effects of administering EGn on embryonic and pregnancy outcomes in CC‐based minimal stimulation cycle IVF are not clear to date. Here, we demonstrated that the cumulative live birthrate for patients with insufficient secretion of endogenous gonadotropin induced by multiple follicle development after CC administration, who were treated with EGn, was comparable to that for the patients treated with clomiphene only, although the rate in the CC‐EGn group was numerically higher than that in the CC group.

We revealed that the combination treatment of CC and EGn resulted in an increase in the number of retrieved oocytes, fertilized oocytes, cleaved embryos, and cryopreserved blastocysts compared with the CC only treatment. These results suggested that the administration of EGn could rescue the insufficiency of endogenous gonadotropins, and more viable embryos could be obtained. However, the blastocyst cryopreservation rate after the combination treatment of CC and gonadotropins was significantly lower than that after treatment with CC only. Furthermore, the correlation between the number of retrieved oocytes and the cumulative live birthrate was weaker in patients treated with CC and EGn than that in patients treated with CC only (OR: 1.193 vs 1.553). Previous studies on rodents reported that ovarian stimulation by administering EGn adversely affected the development of oocytes and embryos.^16^, ^17^, ^18^, ^19^, ^20^ In addition, clinical studies have reported that the live birthrate per oocyte in natural cycles of IVF was higher than that in a controlled ovarian stimulation cycle, suggesting that aggressive ovarian stimulation is likely to yield a high number of oocytes that will not result in a live birth.^21^, ^22^ Thus, the developmental competence of oocytes retrieved from patients treated with CC and EGn possibly may be lower than that of oocytes derived from CC cycles. In the present study, furthermore, EGn was used for patients who exhibited insufficient secretion of endogenous gonadotropin induced by multiple follicles developing after CC administration. On the other hand, the CC group showed sufficient ovarian response to CC alone, meaning they did not need EGn. Therefore, the difference of ovarian response to CC might result in the decreased blastocyst cryopreservation rate in the CC‐EGn group. However, the cumulative live birthrate for patients with insufficient ovarian response to CC who were treated with EGn was statistically comparable to that for patients treated with CC alone. These results suggest that EGn administration to those patients with insufficient secretion of endogenous gonadotropin in the late follicular phase resulted in restoring clinical outcomes even though follicle growth was not sufficient when starting with CC alone.

The main strength of the present study is that we analyzed propensity score matched data. Therefore, the patient characteristics on the initiation day of the oocyte retrieval cycle (day 3) were comparable among the groups. However, there are also several limitations that should be mentioned. First, the retrospective nature of the study is an intrinsic limitation. Although we performed propensity score matching, there might be still some differences in patients’ background, such as the response to CC. Therefore, further randomized controlled clinical trials are required to confirm the effectiveness of gonadotropin supplementation on the cumulative live birthrate in minimal stimulation cycles. Second, the proportion of the usage of each embryo transfer method was different among the two groups. The proportion of the fresh cleaved embryo transfer, frozen cleaved embryo transfer, and frozen blastocyst transfer was 42.1% (85/202), 6.9% (14/202), and 51.0% (103/202) in the CC group, whereas the proportion in the CC‐EGn group was 30.4% (76/250), 6.0% (15/250), and 63.6% (159/250), respectively (*P* = .0236). Previous studies have reported that CC could adversely affect uterine receptivity and pregnancy outcomes during fresh embryo transfer cycles.^10^, ^23^ Furthermore, gonadotropin administration for ovarian stimulation impacts uterine receptivity.^24^, ^25^, ^26^ Therefore, if the embryo transfer method was comparable between the two groups, the results of the statistical analysis might be changed.

In conclusion, the cumulative live birthrate for patients with insufficient secretion of endogenous gonadotropin induced by multiple follicle development after CC administration could be rescued by EGn administration in the late follicular phase after monitoring the follicular growth and serum hormone levels. Therefore, when CC‐based minimal stimulation cycle IVF is performed, the stimulation should be started with CC only, and EGn administration should be scheduled only if insufficient secretion of endogenous gonadotropin is observed in the late follicular phase.

## CONFLICT OF INTEREST

The authors have no conflicts of interest to declare.

## HUMAN RIGHTS, INFORMED CONSENT, AND ETHICAL APPROVAL

The study was a retrospective cohort study approved by the Institutional Review Board of Kato Ladies Clinic (approval number: 19‐28). Written informed consent for retrospective analysis of de‐identified data was obtained from all patients undergoing IVF treatment at the center. All procedures followed were in accordance with the Helsinki Declaration of 1964 and its later amendments.
