# Supplementary material for: Effects of gonadotropin administration on clinical outcomes in clomiphene citrate‐based minimal stimulation cycle IVF
Source: Reprod Med Biol. 2019 Dec 12;19(2):128–34. doi: 10.1002/rmb2.12310 (PMC7138936; doi:10.1002/rmb2.12310)
Supplement: Supplementary file 1 [file RMB2-19-128-s001.docx]

Supplemental Table 1. Cumulative live birth rate per oocyte retrieval, stratified by the number of retrieved oocytes, in clomiphene cycles with/without exogenous gonadotropin

|  | No. of oocytes = 1 | |  | No. of oocytes = 2 | |  | No. of oocytes = 3 | |  | No. of oocytes = 4 | |  | No. of oocytes = 5 | |  | No. of oocytes ≤ 6 | |
| --- | --- | --- | --- | --- | --- | --- | --- | --- | --- | --- | --- | --- | --- | --- | --- | --- | --- |
|  | CC^†^ | CC-EGn^‡^ |  | CC^†^ | CC-EGn^‡^ |  | CC^†^ | CC-EGn^‡^ |  | CC^†^ | CC-EGn^‡^ |  | CC^†^ | CC-EGn^‡^ |  | CC^†^ | CC-EGn^‡^ |
| No. of oocyte retrieval cycles | 69 | 28 |  | 72 | 51 |  | 36 | 41 |  | 14 | 28 |  | 9 | 22 |  | 2 | 44 |
| Women’s age (years) Mean ± SEM | 35.1 ± 0.3 | 35.4 ± 0.5 |  | 35.1 ± 0.3 | 35.1 ± 0.3 |  | 35.3 ± 0.4 | 35.5 ± 0.4 |  | 35.0 ± 0.6 | 34.8 ± 0.5 |  | 36.1 ± 0.7 | 35.6 ± 0.5 |  | 34.5 ± 2.5 | 35.2 ± 0.3 |
| Men’s age (years) Mean ± SEM | 38.8 ± 0.7 | 37.5 ± 0.8 |  | 38.5 ± 0.6 | 38.0 ± 0.7 |  | 36.5 ± 0.5^a^ | 39.1 ± 0.8^b^ |  | 37.2 ± 1.0 | 37.2 ± 0.7 |  | 39.4 ± 0.9 | 39.7 ± 1.0 |  | 39.5 ± 2.5 | 38.5 ± 0.9 |
| No. of matured oocytes Mean ± SEM | 0.9 ± 0.0 | 0.8 ± 0.1 |  | 1.7 ± 0.1 | 1.6 ± 0.1 |  | 2.6 ± 0.1 | 2.4 ± 0.1 |  | 3.2 ± 0.2 | 3.0 ± 0.2 |  | 4.3 ± 0.4 | 3.4 ± 0.3 |  | 5.5 ± 0.5 | 5.8 ± 0.6 |
| No. of fertilized oocytes Mean ± SEM | 0.8 ± 0.0 | 0.8 ± 0.1 |  | 1.6 ± 0.1 | 1.6 ± 0.1 |  | 2.0 ± 0.1 | 2.3 ± 0.1 |  | 2.5 ± 0.3 | 2.5 ± 0.2 |  | 4.0 ± 0.4 | 3.1 ± 0.3 |  | 2.5 ± 1.5 | 5.4 ± 0.5 |
| No. of cleaved embryos Mean ± SEM | 0.8 ± 0.0 | 0.8 ± 0.1 |  | 1.6 ± 0.1 | 1.6 ± 0.1 |  | 2.0 ± 0.1 | 2.2 ± 0.1 |  | 2.5 ± 0.3 | 2.6 ± 0.2 |  | 3.8 ± 0.5 | 3.0 ± 0.4 |  | 2.5 ± 1.5 | 5.3 ± 0.5 |
| Cumulative clinical pregnancy  / Oocyte retrieval cycle (%) | 21  (30.4) | 5  (17.9) |  | 34  (47.2) | 22  (43.1) |  | 18  (50.0) | 21  (51.2) |  | 9  (64.3) | 14  (50.0) |  | 5  (55.6) | 11  (50.0) |  | 1  (50.0) | 27  (61.4) |
| Cumulative ongoing pregnancy  / Oocyte retrieval cycle (%) | 17  (24.6) | 5  (17.9) |  | 32  (44.4) | 20  (39.2) |  | 15  (41.7) | 19  (46.3) |  | 9  (64.3) | 12  (42.9) |  | 4  (44.4) | 10  (45.5) |  | 1  (50.0) | 23  (52.3) |
| Cumulative live birth  / Oocyte retrieval cycle (%) | 15  (21.7) | 4  (14.3) |  | 28  (38.9) | 16  (31.4) |  | 13  (36.1) | 12  (29.3) |  | 8  (57.1) | 11  (39.3) |  | 4  (44.4) | 9  (40.9) |  | 1  (50.0) | 23  (52.3) |
| AORs^§^ for live birth (*P* value) | - | 0.667  (0.5139) |  | - | 0.727  (0.4218) |  | - | 0.897  (0.8354) |  | - | 0.459  (0.2516) |  | - | 0.996  (0.9966) |  | - | 0.675  (0.8013) |

^†^CC, clomiphene; CC-EGn^‡^, clomiphene with exogenous gonadotropins; ^§^AOR, adjusted odds ratio.
